# Supplementary material for: Natural immune boosting biases pertussis infection estimates in seroprevalence studies
Source: Nat Commun. 2025 Oct 6;16:8883. doi: 10.1038/s41467-025-64716-0 (PMC12500921; doi:10.1038/s41467-025-64716-0)
Supplement: Supplementary file 1 — Supplementary Information [file 41467_2025_64716_MOESM1_ESM.pdf]

Natural immune boosting biases pertussis infection estimates in seroprevalence studies

Supplementary Materials

Matthieu Domenech de Cellès[1,*,#], Anabelle Wong[1,2,*], Tine Dalby[3], Pejman Rohani[4,5,6]

1. Max Planck Institute for Infection Biology, Infectious Disease Epidemiology group, Berlin, Germany
2. Institute of Public Health, Charité – Universitätsmedizin Berlin, Berlin, Germany
3. Department of Infectious Disease Epidemiology and Prevention, Statens Serum Institut, Copenhagen, Denmark
4. Odum School of Ecology, University of Georgia, Athens, Georgia, USA
5. Center of Ecology of Infectious Diseases, Athens, Georgia, USA
6. Department of Infectious Diseases, College for Veterinary Medicine, University of Georgia, Athens, Georgia, USA

#Corresponding author: Dr. Matthieu Domenech de Cellès. Address: Max Planck Institute for Infection Biology, Charitéplatz 1, Campus Charité Mitte, 10117 Berlin, Germany. Email: domenech@mpiib-berlin.mpg.de

*These authors contributed equally.

1 Supplementary Methods

1.1 Model formulation

1.1.1 Force of infection

The force of infection in age group *i* was expressed as:

$$\lambda_i = q_i \sum_j M_{ij} \frac{I_1^{(j)} + \theta I_2^{(j)} + \iota}{N_j}$$

Here, q_i represents the age-specific susceptibility to infection, fixed to 0.09 in individuals aged 0–9 yrs, 0.05 in 10–19 yrs, and 0.008 in ≥20 yrs, based on previous fits in Massachusetts, USA [1]. The relative transmissibility of secondary infections was fixed based on the same source ($\theta = 0.99$). The parameters M_{ij} represent the contact rates between age groups *i* and *j* and form the social contact matrix (SCM) *M*, defined on the intensive scale (as per [2]). These SCMs were fixed based on the work of Mistry *et al.* [3]. The parameter ι represents the importation of infections from outside the population, set to a small value ($\iota = 10^{-3}$) to prevent stochastic extinctions. The age-specific population sizes N_j were fixed based on 2010 demographic data in every country, available from Mistry *et al.* [3].

1.1.2 Deterministic equations

The model variables are listed in Table S3. The deterministic variant of the model was described by a set of ordinary differential equations, given below for newborns (age group $i = 0$, the superscript indicates the age group):

$$
\begin{aligned}
\frac{dS_1^{(0)}}{dt} &= bN - (\lambda_0 + \delta_0)S_1^{(0)} \\
\frac{dE_1^{(0)}}{dt} &= \lambda_0 S_1^{(0)} - (\sigma + \delta_0)E_1^{(0)} \\
\frac{dI_1^{(0)}}{dt} &= \sigma E_1^{(0)} - (\gamma + \delta_0)I_1^{(0)} \\
\frac{dS_2^{(0)}}{dt} &= \alpha_R R^{(0)} + \alpha_V V^{(0)} - (\lambda_0 + \delta_0)S_2^{(0)} \\
\frac{dE_2^{(0)}}{dt} &= \lambda_0 S_2^{(0)} - (\sigma + \delta_0)E_2^{(0)} \\
\frac{dI_2^{(0)}}{dt} &= \sigma E_2^{(0)} - (\gamma + \delta_0)I_2^{(0)} \\
\frac{dR_{P,1}^{(0)}}{dt} &= \gamma(I_1^{(0)} + I_2^{(0)}) - (1/t_n + \delta_0)R_{P,1}^{(0)} \\
\frac{dR^{(0)}}{dt} &= R_{P,1}^{(0)}/t_n + R_{P,2}^{(0)}/t_n - (\alpha_R + \rho_R\lambda_0 + \delta_0)R^{(0)} \\
\frac{dR_E^{(0)}}{dt} &= \rho_R\lambda_0 R^{(0)} - (1/t_p + \delta_0)R_E^{(0)} \\
\frac{dR_{P,2}^{(0)}}{dt} &= R_E^{(0)}/t_p - (1/t_n + \delta_0)R_{P,2}^{(0)} \\
\frac{dV^{(0)}}{dt} &= 0 \\
\frac{dV_E^{(0)}}{dt} &= 0 \\
\frac{dV_P^{(0)}}{dt} &= 0
\end{aligned}
$$

In the older age groups ($i \geq 1$), the dynamic was governed by the following set of ODEs:

$$
\begin{aligned}
\frac{dS_1^{(i)}}{dt} &= \delta_{i-1}S_1^{(i-1)} - (\lambda_i + v_i + \delta_i)S_1^{(i)} \\
\frac{dE_1^{(i)}}{dt} &= \delta_{i-1}E_1^{(i-1)} + \lambda_i S_1^{(i)} - (\sigma + \delta_i)E_1^{(i)} \\
\frac{dI_1^{(i)}}{dt} &= \delta_{i-1}I_1^{(i-1)} + \sigma E_1^{(i)} - (\gamma + \delta_i)I_1^{(i)} \\
\frac{dS_2^{(i)}}{dt} &= \delta_{i-1}S_2^{(i-1)} + \alpha_R R^{(i)} + \alpha_V V^{(i)} - (\lambda_i + v_i + \delta_i)S_2^{(i)} \\
\frac{dE_2^{(i)}}{dt} &= \delta_{i-1}E_2^{(i-1)} + \lambda_i S_2^{(i)} - (\sigma + \delta_i)E_2^{(i)} \\
\frac{dI_2^{(i)}}{dt} &= \delta_{i-1}I_2^{(i-1)} + \sigma E_2^{(i)} - (\gamma + \delta_i)I_2^{(i)} \\
\frac{dR_{P,1}^{(i)}}{dt} &= \delta_{i-1}R_{P,1}^{(i-1)} + \gamma(I_1^{(i)} + I_2^{(i)}) - (1/t_n + \delta_i)R_{P,1}^{(i)} \\
\frac{dR^{(i)}}{dt} &= \delta_{i-1}R^{(i-1)} + R_{P,1}^{(i)}/t_n + R_{P,2}^{(i)}/t_n - (\alpha_R + \rho_R\lambda_i + \delta_i)R^{(i)} \\
\frac{dR_E^{(i)}}{dt} &= \delta_{i-1}R_E^{(i-1)} + \rho_R\lambda_i R^{(i)} - (1/t_p + \delta_i)R_E^{(i)} \\
\frac{dR_{P,2}^{(i)}}{dt} &= \delta_{i-1}R_{P,2}^{(i-1)} + R_E^{(i)}/t_p - (1/t_n + \delta_i)R_{P,2}^{(i)} \\
\frac{dV^{(i)}}{dt} &= \delta_{i-1}V^{(i-1)} + v_i(S_1^{(i)} + S_2^{(i)}) + V_P^{(i)}/t_n - (\alpha_V + \rho_V\lambda_i + \delta_i)V^{(i)} \\
\frac{dV_E^{(i)}}{dt} &= \delta_{i-1}V_E^{(i-1)} + \rho_V\lambda_i V^{(i)} - (1/t_p + \delta_i)V_E^{(i)} \\
\frac{dV_P^{(i)}}{dt} &= \delta_{i-1}V_P^{(i-1)} + V_E^{(i)}/t_p - (1/t_n + \delta_i)V_P^{(i)}
\end{aligned}
$$

Here, b represents the *per capita* birth rate and N the total population size; both parameters were fixed based on 2010 demographic data in every country. The δ_i parameters represent aging/mortality rates, which we calculated to reproduce the population age structure (N_i) in every country. The remaining parameters are listed in Table 1.

The parameters v_i represent the effective vaccination rate—*i.e.*, excluding primary vaccine failures—in every age group. These parameters are connected to the age-specific effective vaccination coverage $p_{V,i}$ through the equations:

$$p_{V,i} = \frac{v_i}{v_i+\delta_i} \Rightarrow v_i = \frac{p_{V,i}}{1-p_{V,i}}\delta_i$$

1.1.3 Stochastic formulation

The stochastic implementation used a multinomial modification of the tau-leap algorithm [4] with a fixed time step $\Delta t = 10^{-3}$ years. At every time step, we generated multinomial samples from every source compartment to simulate all possible stochastic transitions in the model (Table S4). We then updated every compartment based on the number of entries and exits simulated for that time step. This process was iterated to simulate the model over time.

1.2 Alternative mode structure

To test the robustness of our results, we considered an alternative model structure where vaccine- and infection-derived immunities were stratified into two stages. For either type of immunity, the corresponding compartments (V and R in the base model) were thus subdivided into two subcompartments (V_1, V_2 and R_1, R_2, respectively). In this extended model, the duration of vaccine immunity (without immune boosting) followed a Gamma distribution with a shape parameter of 2 and a mean of $1/\alpha$, while the duration of infection-derived immunity followed a generalized Erlang, or hypoexponential, distribution (a sum of three exponential distributions with rates $1/t_n$, 2α, and 2α). We further considered two boosting coefficients: ρ for the first stage (V_1 and R_1) and κρ for the second (V_2 and R_2). Here, κ represents the risk of boosting in the second stage relative to the first; we assumed $\kappa \leq 1$ and tested three different values (0, 0.5, and 1). Compared to the previous model, this model allows for a more flexible representation of immune boosting following infection or vaccination. For simplicity, we assumed a deterministic model and a constant population size, omitted the compartments representing exposed states (V_E and R_E) and secondary infections (E_2 and I_2), and ignored age structure (homogeneous model). The corresponding model schematic is displayed in Fig. S5 (bottom panel).

As before, we used a grid search to estimate waning rate values (parameter α) consistent with observed seroprevalences. We considered two target levels of overall seroprevalence, based on two nationwide serosurveys that included all age groups: 10% (Netherlands, 2006–2007 [5]) and 20%

(Australia, 1997–1998 [6]). Because both studies reported seroprevalence estimates for an anti-PT IgG cut-off of 62.5 IU/mL, we fixed the average duration of seropositivity accordingly (t_n=1.9 years). To our knowledge, these two estimates are the highest reported in the vaccine era. We repeated the estimation for two values of ρ (0.5 and 1), which were the most unfavourable to our central hypothesis that serosurveys have low PPV (see Tables 2–3). For completeness, we also repeated the analysis for the homogeneous version of the base model (Fig. S5, top panel). For both models, we assumed a basic reproduction number of 15 and fixed other parameters as in Table 1.

As shown in Table S7, our main results were robust: a combination of low waning immunity and low PPV best explained the target seroprevalence in every scenario. For the homogeneous version of the base model and a target seroprevalence of 20%, the estimated average duration of immunity ranged from 60 years ($\rho = 0.5$) to 150 years ($\rho = 5$). Again, we note the underlying exponential distribution for the duration of vaccine-derived immunity (without boosting), such that these averages translate into a sizeable fraction of vaccinees losing immunity within their lifespan [1,7]. Because of the different distribution assumed for the alternative model (Gamma instead of Exponential), the corresponding estimates were lower but still high, ranging from 30 years ($\rho = 0.5$) to 50–60 years ($\rho = 1$). In keeping with the results from Tables 2–3, the PPV of seropositivity in serosurveys increased as the boosting coefficients decreased but remained low in all scenarios (overall range: 8–25%). Hence, these additional simulations suggest that our main results are relatively unaffected by different modeling assumptions regarding waning immunity and boosting.

2 Supplementary Figures

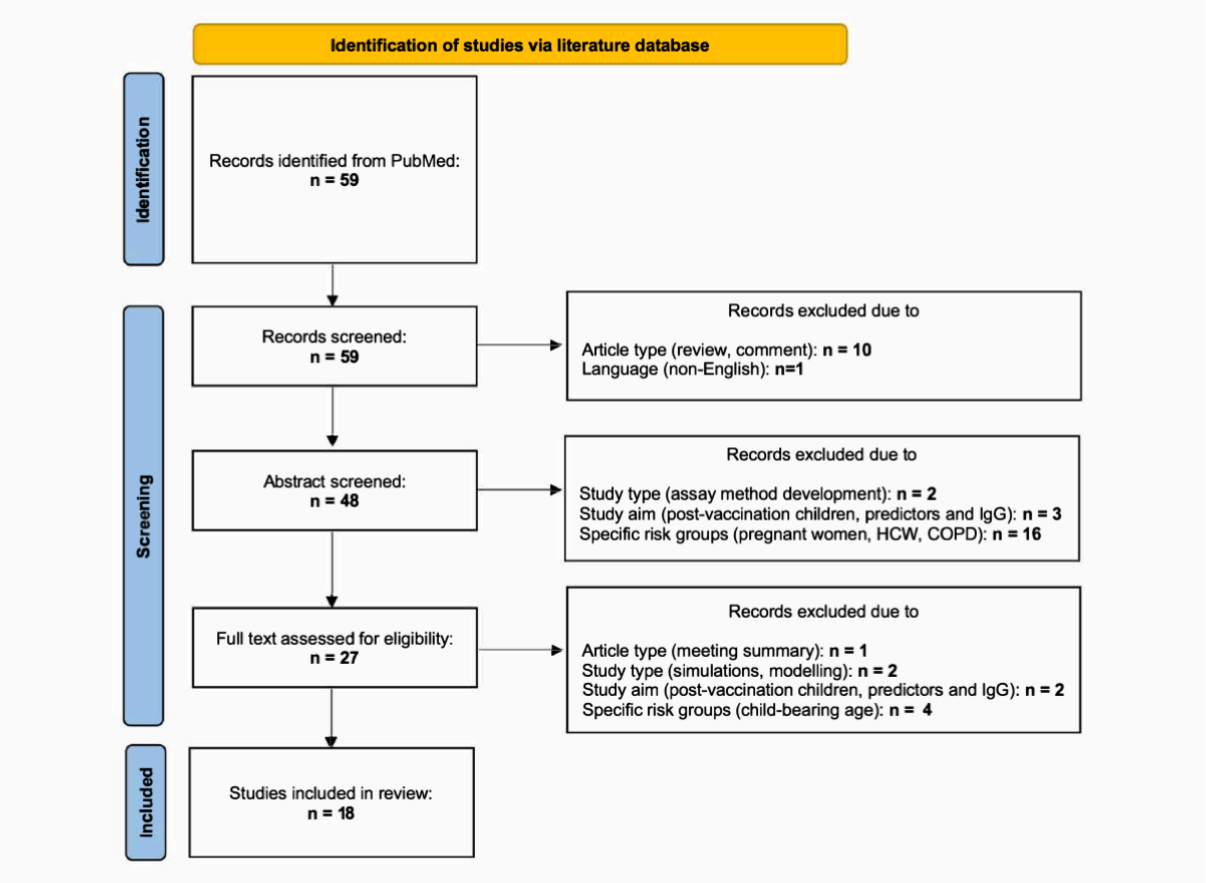

Figure S1: PRISMA flow diagram [8] of the systematic review of pertussis seroprevalence studies.

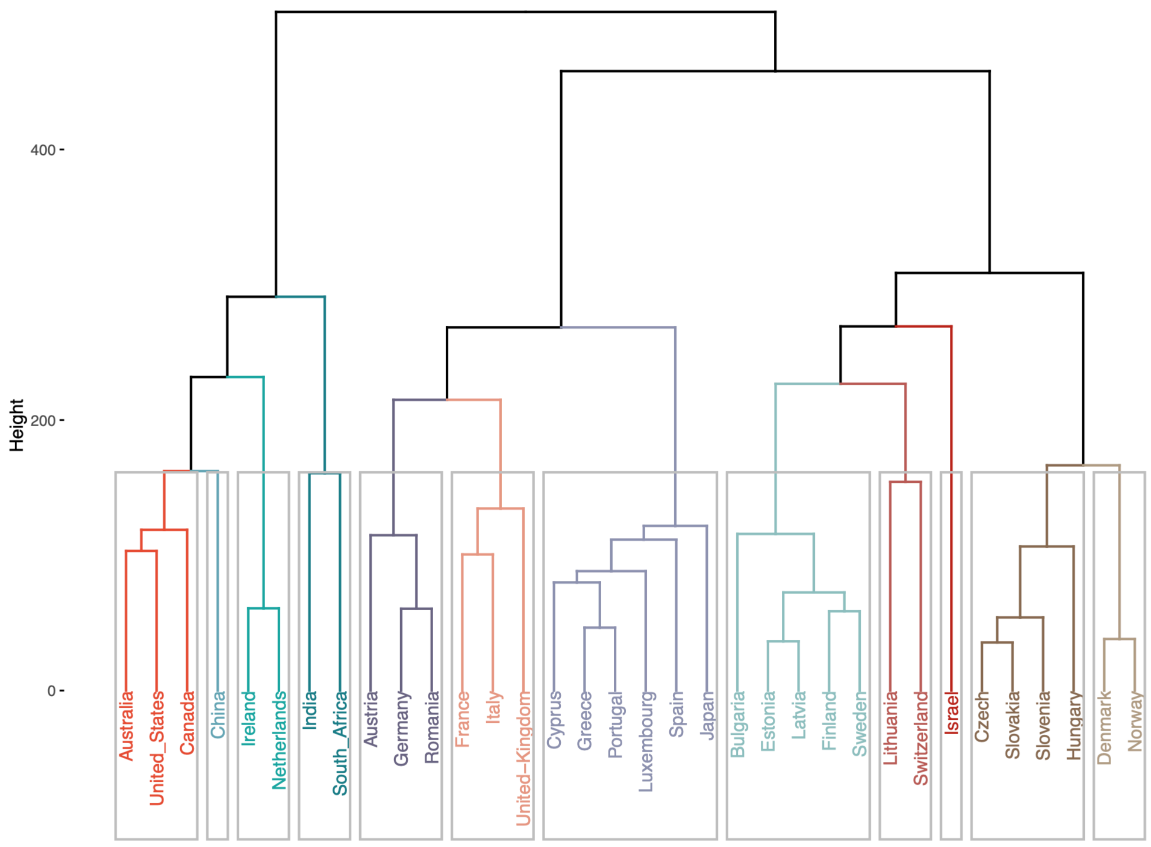

Figure S2: Dendrogram from NGM clustering.

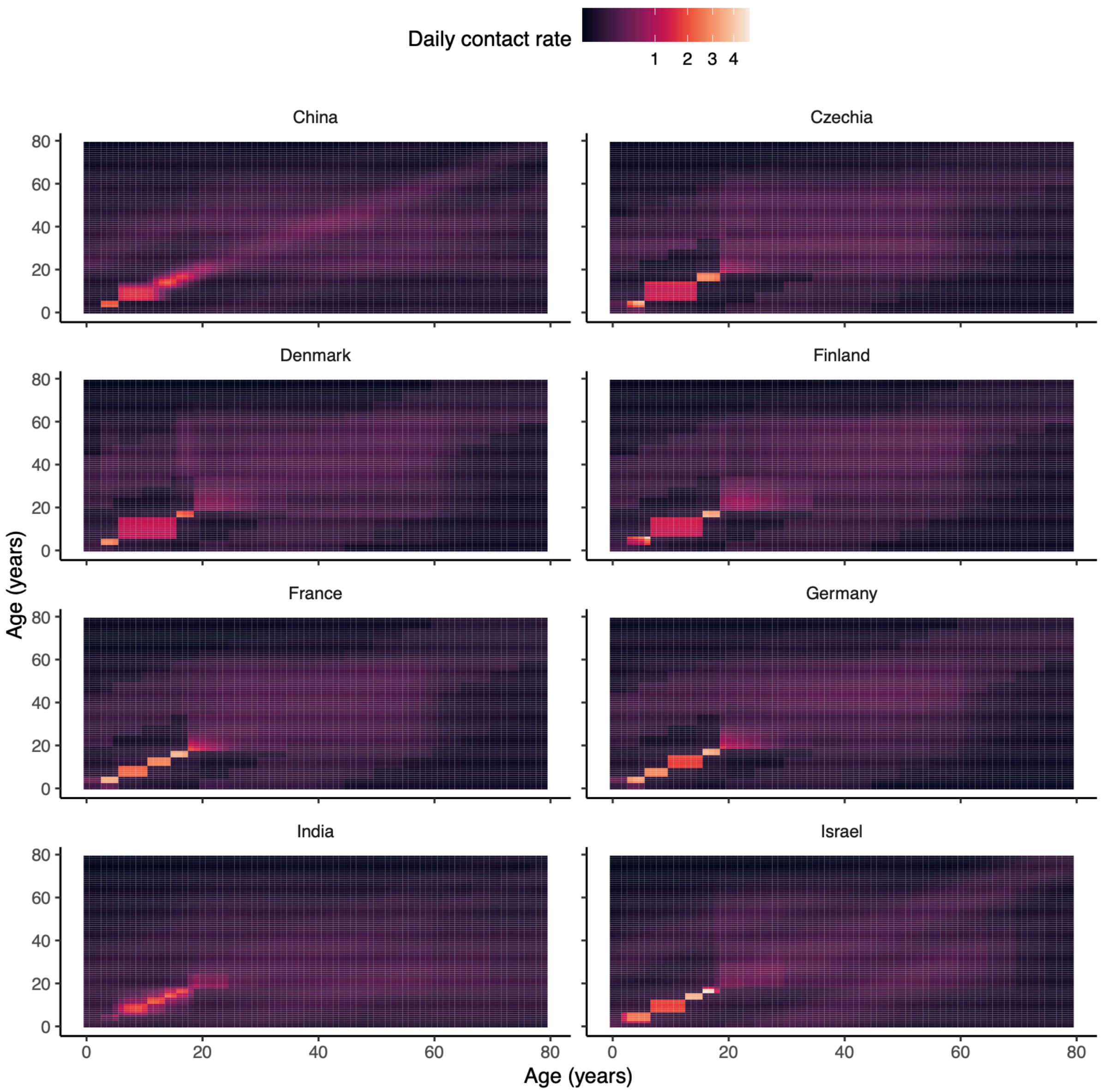

Figure S3: Heatmaps of social contact matrices (*M*) for each country. The color scale is transformed using a square-root function to enhance the visualization of variations at lower contact rates.

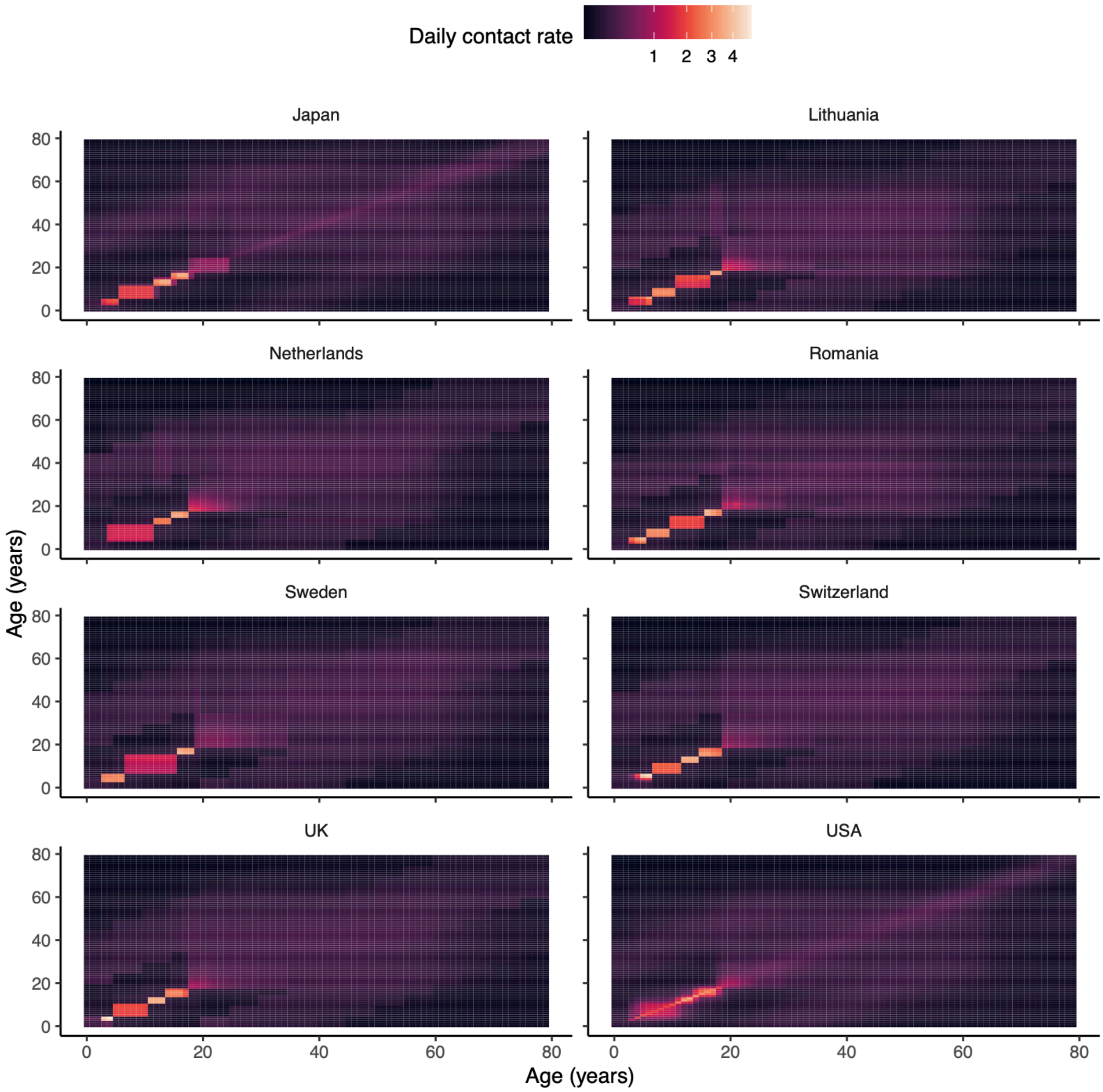

Figure S3 (continued).

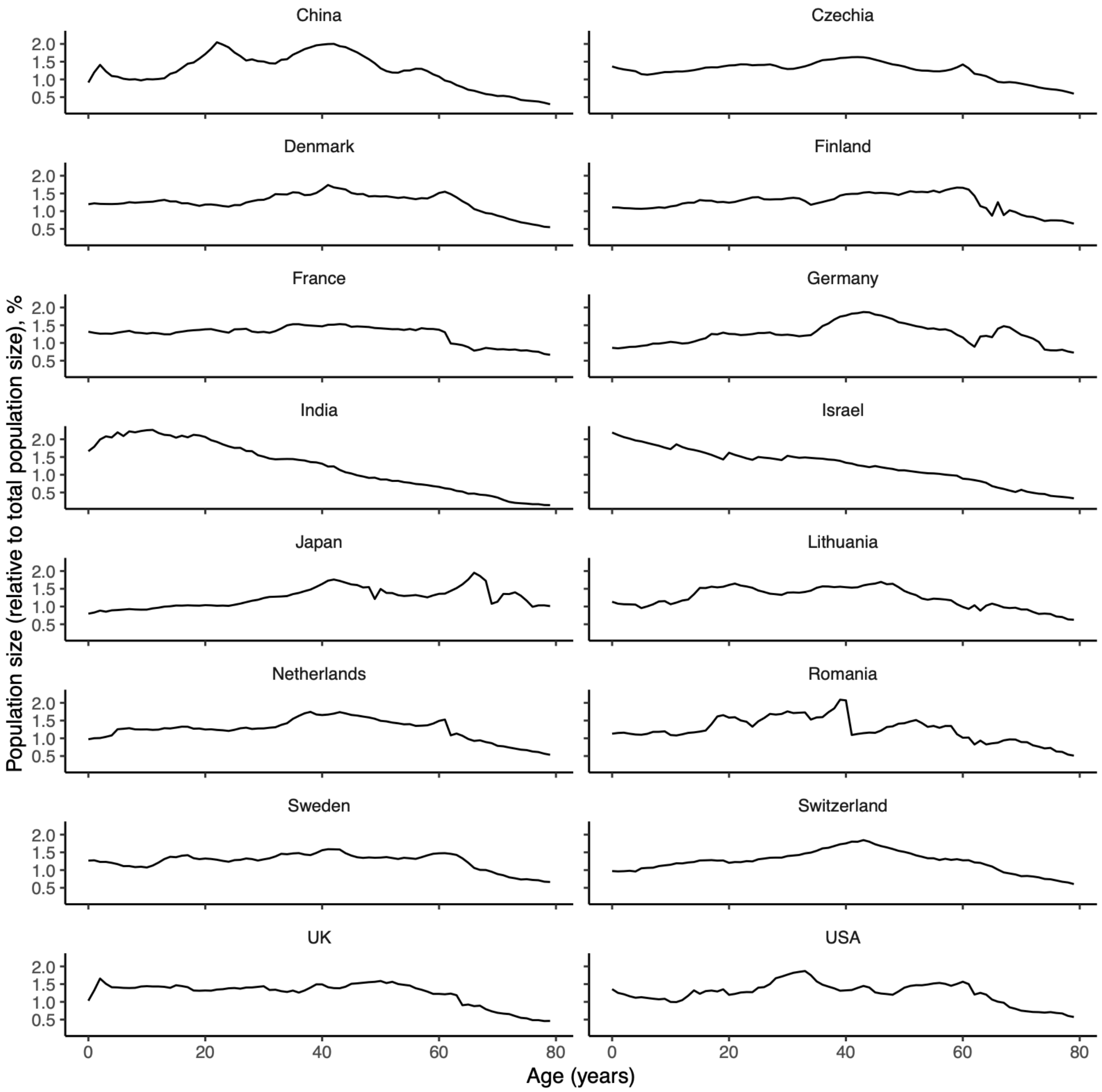

Figure S4: Population structure in the sixteen countries considered for the analysis.

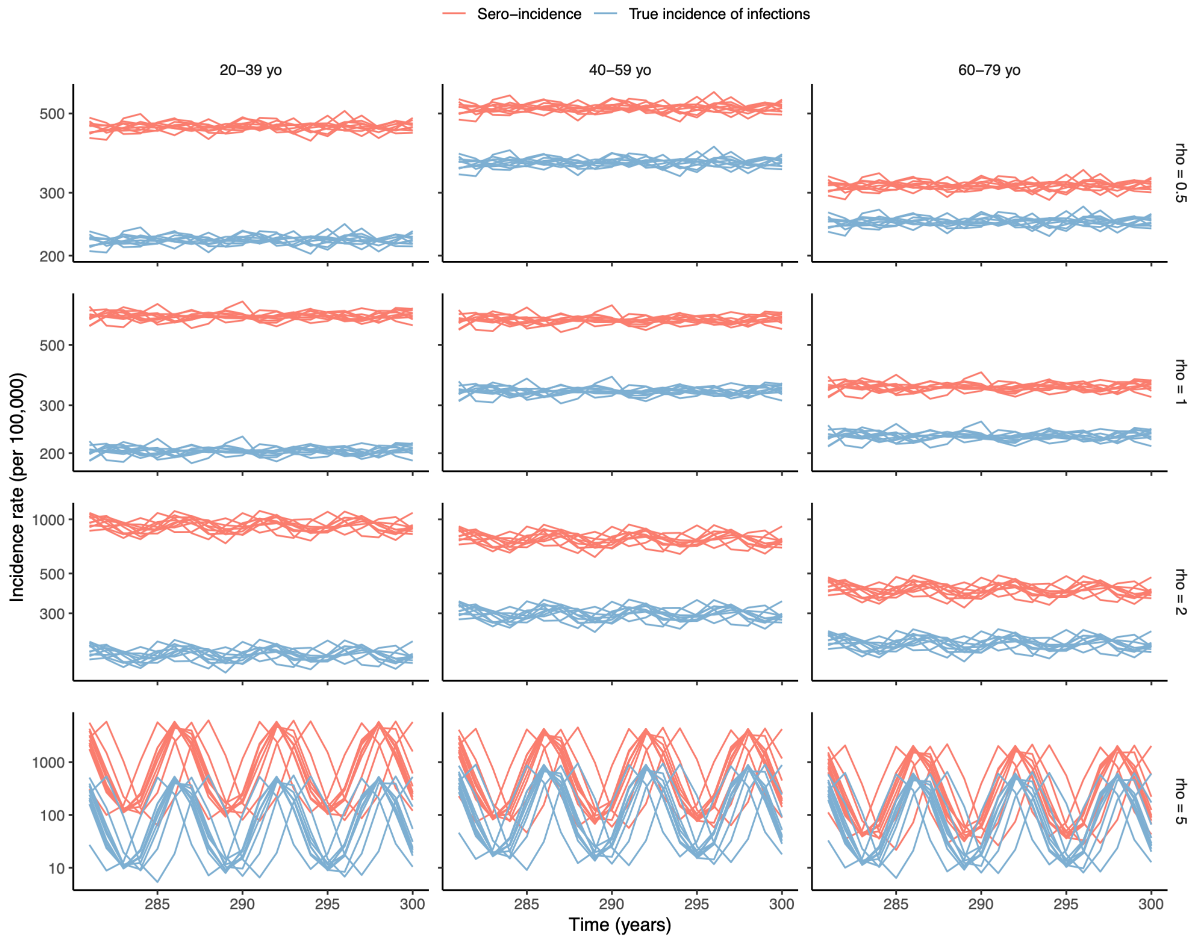

Figure S5: Simulated time series of sero-incidence and true incidence rates in the USA. The *y*-axis values are log-transformed.

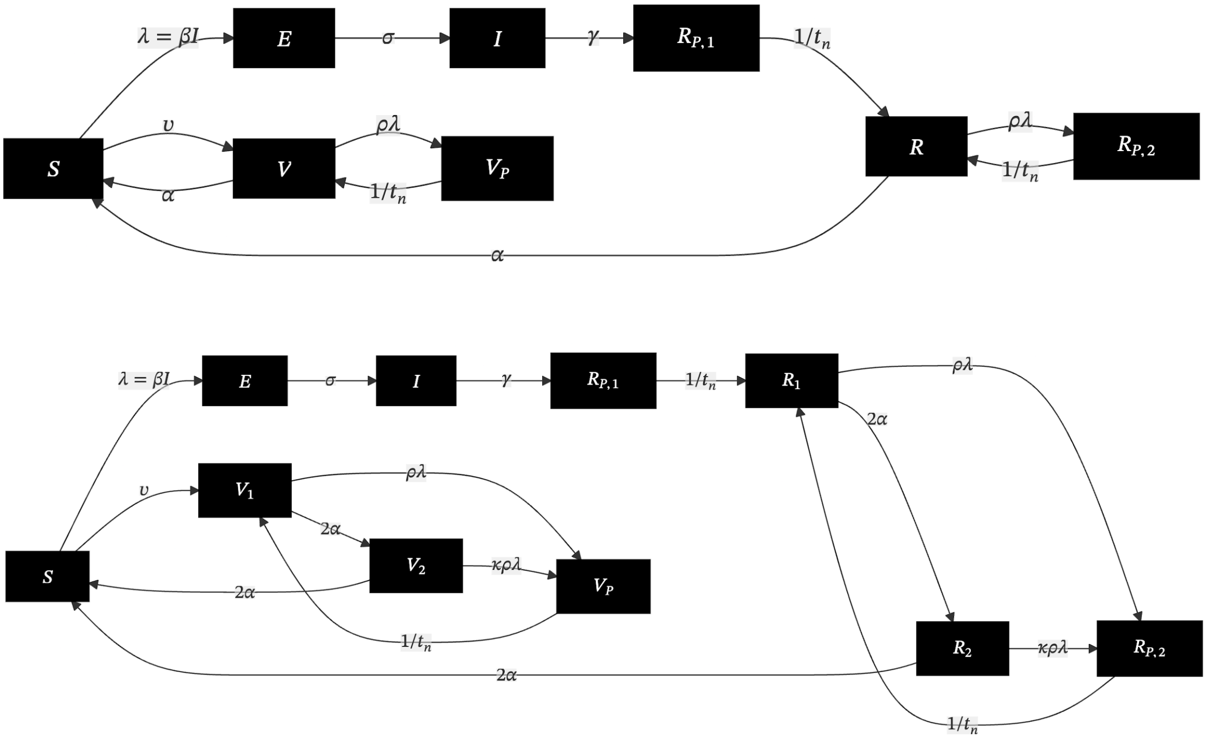

Figure S6: Schematics for the homogeneous versions of the base model (top) and extended model with two boosting coefficients (bottom). NB: For these models, the state variables represent proportions, not numbers.

3 Supplementary Tables

Study	Population	Sample collection	IgG levels	Interpretation
He et al. 2022 [9]	All ages	2020	≤5 IU/mL	Suggestive of no immunity
Liu et al., 2024 [10]	0–3 m – 50 y	2023	≥5 IU/mL	Immune protection against infection
Razafimahatratra et al., 2020 [11]	6 m–15 y	2016	≥5 IU/mL	Immune protection against infection
Pourakbari et al., 2025 [12]	<21 y	2022–2023	>11 U	Suggestive of immunity
Sun et al., 2024 [13]	All ages	2019–2022	≥20 IU/mL	Immune protection against infection
Liu et al., 2021 [14]	1 d – 89 y	2018–2019	≤40 IU/mL	Immune protection against infection
Papagiannis et al., 2022 [15]	30–80+ y	2021–2022	≥50 IU/mL	Immune protection against infection

Table S1: Summary of seroprevalence studies aiming to assess immunity.

Study	Population	Sample collection	IgG levels	Interpretation
Bagordo et al., 2023 [16]	≥6 y	2019–2020	≥100 IU/mL	Recent infection (within last year)
Berber et al., 2021 [17]	40–59 y	2015–2018	≥100 IU/mL	Recent exposure
Chen et al., 2022 [18]	≥15 y	2018–2021	≥100 IU/mL	Recent infection (within 58.6 days)
			≥40 IU/mL	Distant infection (within a few years)
Echaniz-Aviles et al., 2021 [19]	10–25 y	2012	≥94 IU/mL	Recent infection (within last year)
			≥49 IU/mL	Distant infection (within a few years)
Hartanti et al., 2024 [20]	1–14 y	2013–2018	≥100 IU/mL	In age 1–4y: can indicate immune memory (vaccination) or infection<br><br>In age >4 y: high likelihood of infection (within last year)
He et al. 2022 [9]	All ages	2020	≥80 IU/mL	Recent infection in the absence of a vaccination within last year
Kleine et al. 2020 [21]	1–5 y<br>6–19 y	2013<br>2011–2012	≥100 IU/mL	Acute infection or recent vaccination
			[40, 100) IU/mL	Recent infection or vaccination (within last year)
			[5, 40) IU/mL	Distant infection or vaccination (more than a year ago)
Liu et al., 2021 [14]	1 d–89 y	2018–2019	≥100 IU/mL	Acute infection or recent vaccination (within last year)

			[40, 100) IU/mL	Possible course of pertussis
Liu et al., 2024 [10]	All ages	2023	≥100 IU/mL	Acute infection or recent vaccination (within last year)
			≥40 IU/mL	Recent infection (within last year)
Paradowska-Stankiewicz et al., 2021 [22]	5–15 y	2017–2018	IgG ≥100 IU/mL OR IgG ≥40 IU/mL & IgA≥12 IU/mL	Recent infection
Razafimahatratra et al., 2020 [11]	6 m–15 y	2016	≥100 IU/mL	Acute infection or recent vaccination
			[40, 100) IU/mL	Recent exposure
			[5, 40) IU/mL	Distant exposure
Silva et al., 2024 [23]	≥4 y	2015–2018	>120 IU/mL	Recent exposure
			[40, 120) IU/mL	Exposure or vaccination (within last year)
Sun et al., 2024 [13]	All ages	2019–2022	≥80 IU/mL	Recent infection in the absence of a vaccination within last year
Versteegen et al., 2021 [24]	All ages	2016–2017	>100 IU/mL	Recent infection in the absence of a vaccination in the last few years
Wanlapakorn et al. 2024 [25]	All ages	2022–2023	>100 IU/mL	Acute infection or recent vaccination
			40–100 IU/mL	Probable past exposure
			[5, 40) IU/mL	No evidence of acute infection or recent vaccination

Zhang et al., 2024 [26]	All ages	2017–2018	IgG ≥100 IU/mL without recent vaccination<br>OR<br>IgG 40–100 IU/mL & IgA≥12 IU/mL	Probable recent pertussis infection

Table S2: Summary of seroprevalence studies aiming to assess recent exposure or infection.

Variable	Meaning
S_1	Susceptible to primary infection
E_1	Exposed, primary infection
I_1	Infected, primary infection
S_2	Susceptible to secondary infection
E_2	Exposed, secondary infection
I_2	Infected, secondary infection
R	Recovered
R_E	Exposed from recovered state
$R_{P,1}$	Seropositive, after infection
$R_{P,2}$	Seropositive, after immune boost from recovered state
V	Vaccinated
V_E	Exposed from vaccinated state
V_P	Seropositive, after immune boost from vaccinated state

Table S3: List of model variables.

Source compartment	Number of exits	Transition	Rate
$S_1^{(i)}$	3	$S_1^{(i)} \rightarrow S_1^{(i+1)}$	δ_i
		$S_1^{(i)} \rightarrow E_1^{(i)}$	λ_i
		$S_1^{(i)} \rightarrow V^{(i)}$	v_i
$E_1^{(i)}$	2	$E_1^{(i)} \rightarrow E_1^{(i+1)}$	δ_i
		$E_1^{(i)} \rightarrow I_1^{(i)}$	σ
$I_1^{(i)}$	2	$I_1^{(i)} \rightarrow I_1^{(i+1)}$	δ_i
		$I_1^{(i)} \rightarrow R_{P,1}^{(i)}$	γ
$S_2^{(i)}$	3	$S_2^{(i)} \rightarrow S_2^{(i+1)}$	δ_i
		$S_2^{(i)} \rightarrow E_2^{(i)}$	λ_i
		$S_2^{(i)} \rightarrow V^{(i)}$	v_i
$E_2^{(i)}$	2	$E_2^{(i)} \rightarrow E_2^{(i+1)}$	δ_i
		$E_2^{(i)} \rightarrow I_2^{(i)}$	σ
$I_2^{(i)}$	2	$I_2^{(i)} \rightarrow I_2^{(i+1)}$	δ_i
		$I_2^{(i)} \rightarrow R_{P,1}^{(i)}$	γ
$R^{(i)}$	3	$R^{(i)} \rightarrow R^{(i+1)}$	δ_i
		$R^{(i)} \rightarrow S_2^{(i)}$	α_R
		$R^{(i)} \rightarrow R_E^{(i)}$	$\rho_R \lambda_i$
$R_E^{(i)}$	2	$R_E^{(i)} \rightarrow R_E^{(i+1)}$	δ_i
		$R_E^{(i)} \rightarrow R_{P,2}^{(i)}$	$1/t_p$

$R_{P,1}^{(i)}$	2	$R_{P,1}^{(i)} \rightarrow R_{P,1}^{(i+1)}$	δ_i
		$R_{P,1}^{(i)} \rightarrow R^{(i)}$	$1/t_n$
$R_{P,2}^{(i)}$	2	$R_{P,2}^{(i)} \rightarrow R_{P,2}^{(i+1)}$	δ_i
		$R_{P,2}^{(i)} \rightarrow R^{(i)}$	$1/t_n$
$V^{(i)}$	3	$V^{(i)} \rightarrow V^{(i+1)}$	δ_i
		$V^{(i)} \rightarrow S_2^{(i)}$	α_V
		$V^{(i)} \rightarrow V_E^{(i)}$	$\rho_V \lambda_i$
$V_E^{(i)}$	2	$V_E^{(i)} \rightarrow V_E^{(i+1)}$	δ_i
		$V_E^{(i)} \rightarrow V_P^{(i)}$	$1/t_p$
$V_P^{(i)}$	2	$V_P^{(i)} \rightarrow V_P^{(i+1)}$	δ_i
		$V_P^{(i)} \rightarrow V^{(i)}$	$1/t_n$

Table S4: List of transitions for implementing the stochastic model variant.

Serosurvey	Country	Year(s) of serosurvey	Reason for exclusion
Pebody *et al.* [27]	England & Wales	1996	Low vaccine coverage during 1970–1989 [27]
	Italy	1996	Low vaccine coverage during 1970–1999 [27]
	West Germany	1995	Low vaccine coverage until the 1990s [27]
Wehlin *et al.* [28]	Belgium	2012–13	No SCM data from Mistry *et al.* [3]
	Malta	2012	No SCM data from Mistry *et al.* [3]
	Poland	2010–11	No SCM data from Mistry *et al.* [3]
	Denmark	2012–13	Switch to aP in 1997 [29]
	Finland	2011–12	Switch to aP in 2005 [29]
	Greece	2012	Switch to aP in 1997 [30]
	Hungary	2012	Switch to aP in 2006 [31]
	Italy	2012–13	Switch to aP in 1995 [29]
	Norway	2011–13	Switch to aP in 1998 [29]
	Portugal	2012	Switch to aP in 2006 [32]
	Spain	2011	Switch to aP in 2005 [29]
	Sweden	2012	Switch to aP in 1996 [29]

Table S5: Countries excluded from the analysis of seroprevalence data.

Country	Age of first dose	Vaccine coverage*	Age(s) of booster doses	wP start year	Year of serosurvey	Simulated period (years after wP start)**	Sources
Finland	3 mo	~99%	2 yr	1952	1996	44±5=39–49	[27,29,33]
France	2 mo	~85–95%	1 yr	1959	1998	39±5=34–44	[27,29,34]
East Germany	3 mo	85–~95%	3 yr	1964	1995	31±5=26–36	[27,35]
Lithuania	3 mo	93–97%	1 yr	1961	2012	38±5=33–43	[31]
Netherlands	3 mo	96–97%	1 yr	1957	1995	51±5=46–56	[27,29]
Romania	2 mo	95–99%	1 yr, 3 yr	1961	2010–11	50±5=45–55	[31]

Table S6: Characteristics of countries included in the analysis of seroprevalence data. *Data represent the coverage with four doses in Lithuania and Romania (source: [31]), with three doses in the other countries (source: [27]). Based on these data, we assumed, for simplicity, an effective vaccine coverage of 90% for all doses, corresponding to a 95% vaccine coverage and a 5% probability of primary vaccine failure [1]. **We defined a simulated period of ±5 years after wP start to take into account potential uncertainties in the start date and initial coverage of wP programs.

Model	Target seroprevalence	ρ (fixed)	κ (fixed)	α^{-1} (estimated, years)	PPV (%)
Base (Fig. S5, top schematic)	10%	0.5	—	250	14
		1		>500	<8
	20%	0.5		60	15
		1		150	8
Extended (Fig. S5, bottom schematic)	10%	0.5	0	90	21
			0.5	100	15
			1	95	14
		1	0	100	12
			0.5	>500	<8
			1	>500	<7
	20%	0.5	0	30	25
			0.5	30	18
			1	30	15
		1	0	60	14
			0.5	55	9
			1	50	8

Table S7: Estimates of the average duration of immunity for the homogeneous base and extended models. Parameters: ρ, boosting coefficient during the first vaccinated/recovered stage; κρ, boosting coefficient during the second vaccinated/recovered stage; α^{-1}, average duration of vaccine/infection-derived immunity. PPV: positive predictive value of seropositivity in serosurveys. All models were fitted to reach a target seroprevalence of 10% or 20% at equilibrium; the associated error was almost null in all scenarios.

4 Supplementary References

1. Domenech de Cellès M, Magpantay FMG, King AA, Rohani P. The impact of past vaccination coverage and immunity on pertussis resurgence. Sci Transl Med. 2018;10. doi:10.1126/scitranslmed.aaj1748

2. Arregui S, Aleta A, Sanz J, Moreno Y. Projecting social contact matrices to different demographic structures. PLoS Comput Biol. 2018;14: e1006638. doi:10.1371/journal.pcbi.1006638

3. Mistry D, Litvinova M, Pastore Y Piontti A, Chinazzi M, Fumanelli L, Gomes MFC, et al. Inferring high-resolution human mixing patterns for disease modeling. Nat Commun. 2021;12: 323. doi:10.1038/s41467-020-20544-y

4. He D, Ionides EL, King AA. Plug-and-play inference for disease dynamics: measles in large and small populations as a case study. J R Soc Interface. 2010;7: 271–283. doi:10.1098/rsif.2009.0151

5. de Greeff SC, de Melker HE, van Gageldonk PGM, Schellekens JFP, van der Klis FRM, Mollema L, et al. Seroprevalence of pertussis in The Netherlands: evidence for increased circulation of Bordetella pertussis. PLoS One. 2010;5: e14183. doi:10.1371/journal.pone.0014183

6. Campbell P, McIntyre P, Quinn H, Hueston L, Gilbert GL, McVernon J. Increased population prevalence of low pertussis toxin antibody levels in young children preceding a record pertussis epidemic in Australia. PLoS One. 2012;7: e35874. doi:10.1371/journal.pone.0035874

7. Wearing HJ, Rohani P. Estimating the duration of pertussis immunity using epidemiological signatures. PLoS Pathog. 2009;5: e1000647. doi:10.1371/journal.ppat.1000647

8. Page MJ, McKenzie JE, Bossuyt PM, Boutron I, Hoffmann TC, Mulrow CD, et al. The PRISMA 2020 statement: an updated guideline for reporting systematic reviews. BMJ. 2021;372: n71. doi:10.1136/bmj.n71

9. He H, Zhu Y, Jin M, Zhou Y, Tang X, Yan R, et al. The decline in immunity and circulation of pertussis among Chinese population during the COVID-19 pandemic: A cross-sectional sero-epidemiological study. Vaccine. 2022;40: 6956–6962. doi:10.1016/j.vaccine.2022.10.020

10. Liu Y, Zhang C, Wang Y, Luo X, Liu G, Zhang Z, et al. Seroepidemiology of pertussis in Huzhou: A population-based, cross-sectional study. PLoS One. 2024;19: e0303508. doi:10.1371/journal.pone.0303508

11. Razafimahatratra SL, Wesolowski A, Rafetrarivony L, Heraud J-M, Jones FK, Cauchemez S, et al. Seroprevalence of pertussis in Madagascar and implications for vaccination. Epidemiol Infect. 2020;148: e283. doi:10.1017/S0950268820002800

12. Pourakbari B, Mahmoudi S, Sajedi Moghaddam S, Jafari E, Azizian R, Sotoudeh M, et al. Evaluation of anti-Pertussis antibody levels in Iranian infants and children: Is it time to include booster acellular Pertussis Vaccines in the immunization schedule? Vaccine. 2025;48: 126736. doi:10.1016/j.vaccine.2025.126736

13. Sun X, Zhang T, Sun J, Zhou J, Chen Q, Jia C, et al. The seroepidemiology of immunoglobulin G antibodies against pertussis toxin and filamentous hemagglutinin in the east of China during the COVID-19 pandemic. Hum Vaccin Immunother. 2024;20: 2331438. doi:10.1080/21645515.2024.2331438

14. Liu D, Cheng X, Wei S, Yuan L, Chen C, Yao K. Decline of serologic immunity to diphtheria, tetanus and pertussis with age suggested a full life vaccination in mainland China. Hum Vaccin Immunother. 2021;17: 1757–1762. doi:10.1080/21645515.2020.1840253

15. Papagiannis D, Rachiotis G, Mariolis A, Zafiriou E, Gourgoulianis KI. Vaccination coverage of the elderly in Greece: A cross-sectional nationwide study. Can J Infect Dis Med Microbiol. 2020;2020: 5459793. doi:10.1155/2020/5459793

16. Bagordo F, Grassi T, Savio M, Rota MC, Baldovin T, Vicentini C, et al. Assessment of Pertussis Underreporting in Italy. J Clin Med. 2023;12. doi:10.3390/jcm12051732

17. Berbers G, van Gageldonk P, Kassteele J van de, Wiedermann U, Desombere I, Dalby T, et al. Circulation of pertussis and poor protection against diphtheria among middle-aged adults in 18 European countries. Nat Commun. 2021;12: 2871. doi:10.1038/s41467-021-23114-y

18. Chen Q, Wang W, Shi X, Xu Y, Zhu Y, Wu Y, et al. Seroepidemiology of pertussis in the east of China: Estimates of incidence of infection in adolescents and adults pre- and post-COVID-19. Front Public Health. 2022;10: 1054617. doi:10.3389/fpubh.2022.1054617

19. Echaniz-Aviles G, García-Cisneros S, Sánchez-Alemán MA, Olamendi-Portugal M, Romero-Martinez M, Deantonio R, et al. Estimating Bordetella pertussis seroprevalence in adolescents and young adults in Mexico using the 2012 National Health and Nutrition Survey (ENSANUT). Vaccine. 2021;39: 5839–5844. doi:10.1016/j.vaccine.2021.08.063

20. Hartanti MD, Panjaitan NSD, Sunarno S, Ningrum N, Hasugian AR, Dewi RM, et al. Seroprevalence of Bordetella pertussis infection in children 1-14 years old: Indonesia basic health research (Riskesdas) 2013 and 2018 data. PLoS One. 2024;19: e0311362. doi:10.1371/journal.pone.0311362

21. Kleine D, Billamay S, Chanthavilay P, Mongkhoune S, Keokhamphoui C, Souksakhone C, et al. Pertussis in Lao PDR: Seroprevalence and disease. Int J Infect Dis. 2020;95: 282–287. doi:10.1016/j.ijid.2020.03.074

22. Paradowska-Stankiewicz I, Rumik A, Bogusz J, Zbrzeźniak J, Rastawicki W, Śmietańska K, et al. Duration of protection against Bordetella pertussis infection elicited by whole-cell and acellular vaccine priming in Polish children and adolescents. Vaccine. 2021;39: 6067–6073. doi:10.1016/j.vaccine.2021.08.105

23. Silva EP, Trentini M, Rodriguez D, Kanno AI, Gomes FMS, Valente MH, et al. Seroprevalence study reveals pertussis underreporting in Brazil and calls for adolescent/young adult boosting: mouse model demonstrates immunity restoration. Front Immunol. 2024;15: 1472157. doi:10.3389/fimmu.2024.1472157

24. Versteegen P, Berbers GAM, Smits G, Sanders EAM, van der Klis FRM, de Melker HE, et al. More than 10 years after introduction of an acellular pertussis vaccine in infancy: a cross-sectional serosurvey of pertussis in the Netherlands. Lancet Reg Health Eur. 2021;10: 100196. doi:10.1016/j.lanepe.2021.100196

25. Wanlapakorn N, Suntronwong N, Kanokudom S, Assawakosri S, Vichaiwattana P, Klinfueng S, et al. Seroprevalence of antibodies against diphtheria, tetanus, and pertussis across various age groups during the post-COVID-19 pandemic period in Chonburi Province, Thailand. Heliyon. 2024;10: e39889. doi:10.1016/j.heliyon.2024.e39889

26. Zhang Z, Wang Q, Zhu Q, Bai S, Liu Y, Ren J, et al. Seroepidemiology of pertussis immunity in five provinces of China: A population-based, cross-sectional study. Hum Vaccin Immunother. 2024;20: 2417532. doi:10.1080/21645515.2024.2417532

27. Pebody RG, Gay NJ, Giammanco A, Baron S, Schellekens J, Tischer A, et al. The seroepidemiology of Bordetella pertussis infection in Western Europe. Epidemiol Infect. 2005;133: 159–171. doi:10.1017/s0950268804003012

28. Wehlin L, Ljungman M, Kühlmann-Berenzon S, Galanis I, Huygen K, Pierard D, et al. Pertussis seroprevalence among adults of reproductive age (20-39 years) in fourteen European countries. APMIS. 2021;129: 556–565. doi:10.1111/apm.13165

29. Wong A, Opinel A, Combes SJ-B, Toubiana J, Brisse S. Determining factors for pertussis vaccination policy: A study in five EU countries. Vaccines (Basel). 2020;8: 46. doi:10.3390/vaccines8010046

30. Theodoridou M, Dargenta G, Aptouramani M, Papastergiou P, Katsiaflaka A, Theodoridou K, et al. Pertussis Epidemiology in Greece and Emerging Risk Groups during the Vaccination Era (1980–2008). Adv Prev Med. 2012;2012. doi:10.1155/2012/303846

31. Heininger U, André P, Chlibek R, Kristufkova Z, Kutsar K, Mangarov A, et al. Comparative epidemiologic characteristics of pertussis in 10 Central and Eastern European countries, 2000-2013. PLoS One. 2016;11: e0155949. doi:10.1371/journal.pone.0155949

32. Gama de Sousa S, Barros H. Pertussis in Portugal - time for a new strategy. Rev Port Pneumol. 2010;16: 573–588. doi:10.1016/s2173-5115(10)70060-9

33. Elomaa A, Advani A, Donnelly D, Antila M, Mertsola J, Hallander H, et al. Strain variation among Bordetella pertussis isolates in finland, where the whole-cell pertussis vaccine has been used for 50 years. J Clin Microbiol. 2005;43: 3681–3687. doi:10.1128/JCM.43.8.3681-3687.2005

34. Blanchard E, Chavade D, de Wazières B, Bakhache P, Fumet T, Guiso N. Pertussis vaccination in adults in France: Overview and suggestions for improvement. Infect Dis Now. 2024;54: 104961. doi:10.1016/j.idnow.2024.104961

35. Hellenbrand W, Beier D, Jensen E, Littmann M, Meyer C, Oppermann H, et al. The epidemiology of pertussis in Germany: past and present. BMC Infect Dis. 2009;9: 22. doi:10.1186/1471-2334-9-22

PRISMA 2020 Checklist

Section and Topic	Item #	Checklist item	Location where item is reported
**TITLE**			
Title	1	Identify the report as a systematic review.	
**ABSTRACT**			
Abstract	2	See the PRISMA 2020 for Abstracts checklist.	
**INTRODUCTION**			
Rationale	3	Describe the rationale for the review in the context of existing knowledge.	Methods, Results
Objectives	4	Provide an explicit statement of the objective(s) or question(s) the review addresses.	Methods, Results
**METHODS**			
Eligibility criteria	5	Specify the inclusion and exclusion criteria for the review and how studies were grouped for the syntheses.	Methods, Results, Fig S1
Information sources	6	Specify all databases, registers, websites, organisations, reference lists and other sources searched or consulted to identify studies. Specify the date when each source was last searched or consulted.	Methods, Fig S1
Search strategy	7	Present the full search strategies for all databases, registers and websites, including any filters and limits used.	Methods
Selection process	8	Specify the methods used to decide whether a study met the inclusion criteria of the review, including how many reviewers screened each record and each report retrieved, whether they worked independently, and if applicable, details of automation tools used in the process.	Methods
Data collection process	9	Specify the methods used to collect data from reports, including how many reviewers collected data from each report, whether they worked independently, any processes for obtaining or confirming data from study investigators, and if applicable, details of automation tools used in the process.	Methods
Data items	10a	List and define all outcomes for which data were sought. Specify whether all results that were compatible with each outcome domain in each study were sought (e.g. for all measures, time points, analyses), and if not, the methods used to decide which results to collect.	
	10b	List and define all other variables for which data were sought (e.g. participant and intervention characteristics, funding sources). Describe any assumptions made about any missing or unclear information.	
Study risk of bias assessment	11	Specify the methods used to assess risk of bias in the included studies, including details of the tool(s) used, how many reviewers assessed each study and whether they worked independently, and if applicable, details of automation tools used in the process.	
Effect measures	12	Specify for each outcome the effect measure(s) (e.g. risk ratio, mean difference) used in the synthesis or presentation of results.	
Synthesis methods	13a	Describe the processes used to decide which studies were eligible for each synthesis (e.g. tabulating the study intervention characteristics and comparing against the planned groups for each synthesis (item #5)).	
	13b	Describe any methods required to prepare the data for presentation or synthesis, such as handling of missing summary statistics, or data conversions.	
	13c	Describe any methods used to tabulate or visually display results of individual studies and syntheses.	
	13d	Describe any methods used to synthesize results and provide a rationale for the choice(s). If meta-analysis was performed, describe the model(s), method(s) to identify the presence and extent of statistical heterogeneity, and software package(s) used.	
	13e	Describe any methods used to explore possible causes of heterogeneity among study results (e.g. subgroup analysis, meta-regression).	

PRISMA

PRISMA 2020 Checklist

Section and Topic	Item #	Checklist item	Location where item is reported
	13f	Describe any sensitivity analyses conducted to assess robustness of the synthesized results.	
Reporting bias assessment	14	Describe any methods used to assess risk of bias due to missing results in a synthesis (arising from reporting biases).	
Certainty assessment	15	Describe any methods used to assess certainty (or confidence) in the body of evidence for an outcome.	
**RESULTS**			
Study selection	16a	Describe the results of the search and selection process, from the number of records identified in the search to the number of studies included in the review, ideally using a flow diagram.	Results, Fig S1
	16b	Cite studies that might appear to meet the inclusion criteria, but which were excluded, and explain why they were excluded.	
Study characteristics	17	Cite each included study and present its characteristics.	Table S1, Table S2
Risk of bias in studies	18	Present assessments of risk of bias for each included study.	
Results of individual studies	19	For all outcomes, present, for each study: (a) summary statistics for each group (where appropriate) and (b) an effect estimate and its precision (e.g. confidence/credible interval), ideally using structured tables or plots.	Table S1, Table S2
Results of syntheses	20a	For each synthesis, briefly summarise the characteristics and risk of bias among contributing studies.	
	20b	Present results of all statistical syntheses conducted. If meta-analysis was done, present for each the summary estimate and its precision (e.g. confidence/credible interval) and measures of statistical heterogeneity. If comparing groups, describe the direction of the effect.	
	20c	Present results of all investigations of possible causes of heterogeneity among study results.	
	20d	Present results of all sensitivity analyses conducted to assess the robustness of the synthesized results.	
Reporting biases	21	Present assessments of risk of bias due to missing results (arising from reporting biases) for each synthesis assessed.	
Certainty of evidence	22	Present assessments of certainty (or confidence) in the body of evidence for each outcome assessed.	
**DISCUSSION**			
Discussion	23a	Provide a general interpretation of the results in the context of other evidence.	Results
	23b	Discuss any limitations of the evidence included in the review.	Results
	23c	Discuss any limitations of the review processes used.	
	23d	Discuss implications of the results for practice, policy, and future research.	Results
**OTHER INFORMATION**			
Registration and protocol	24a	Provide registration information for the review, including register name and registration number, or state that the review was not registered.	
	24b	Indicate where the review protocol can be accessed, or state that a protocol was not prepared.	
	24c	Describe and explain any amendments to information provided at registration or in the protocol.	
Support	25	Describe sources of financial or non-financial support for the review, and the role of the funders or sponsors in the review.	
Competing interests	26	Declare any competing interests of review authors.	Competing interests
Availability of	27	Report which of the following are publicly available and where they can be found: template data collection forms; data extracted from included	

PRISMA 2020 Checklist

Section and Topic	Ite m #	Checklist item	Location where item is reported
data, code and other materials		studies; data used for all analyses; analytic code; any other materials used in the review.	

From: Page MJ, McKenzie JE, Bossuyt PM, Boutron I, Hoffmann TC, Mulrow CD, et al. The PRISMA 2020 statement: an updated guideline for reporting systematic reviews. BMJ 2021;372:n71. doi: 10.1136/bmj.n71.
This work is licensed under CC BY 4.0. To view a copy of this license, visit https://creativecommons.org/licenses/by/4.0/
